# Bézier Curves-Based Optimal Trajectory Design for Multirotor UAVs with Any-Angle Pathfinding Algorithms

**DOI:** 10.3390/s21072460

**Published:** 2021-04-02

**Authors:** Haitham AL Satai, Musaddak M. Abdul Zahra, Zaid I. Rasool, Ridhab Sami Abd-Ali, Catalin I. Pruncu

**Affiliations:** 1 School of Electronics Information and Electrical Engineering, Shanghai Jiao Tong University, Shanghai 200240, China; haithamalsatai@gmail.com; 2 Computer Techniques Engineering Department, Al-Mustaqbal University College, Babylon 51001, Iraq; musaddaqmahir@mustaqbal-college.edu.iq (M.M.A.Z.); Zaid.Ibrahim@mustaqbal-college.edu.iq (Z.I.R.); RidhabSami@mustaqbal-college.edu.iq (R.S.A.-A.); 3 Electrical Engineering Department, College of Engineering, University of Babylon, Babylon 51001, Iraq; 4 Department of Mechanical Engineering, Imperial Colle London, Exhibition Rd., London SW7 2AZ, UK; 5 Design, Manufacturing & Engineering Management, University of Strathclyde, Glasgow G1 1XJ, Scotland, UK

**Keywords:** path planning, trajectory planning, Basic Theta*, Lazy Theta*, Phi*, Bézier curves

## Abstract

Multirotor Unmanned Aerial Vehicles (UAVs) play an imperative role in many real-world applications in a variety of scenarios characterized by a high density of obstacles with different heights. Due to the complicated operation areas of UAVs and complex constraints associated with the assigned mission, there should be a suitable path to fly. Therefore, the most relevant challenge is how to plan a flyable path for a UAV without collisions with obstacles. This paper demonstrates how a flyable and continuous trajectory was constructed by using any-angle pathfinding algorithms, which are Basic Theta*, Lazy Theta*, and Phi* algorithms for a multirotor UAV in a cluttered environment. The three algorithms were modified by adopting a modified cost function during their implementation that considers the elevation of nodes. First, suitable paths are generated by using a modified version of the three algorithms. After that, four Bézier curves-based approaches are proposed to smooth the generated paths to be converted to flyable paths (trajectories). To determine the most suitable approach, particularly when searching for an optimal and collision-free trajectory design, an innovative evaluation process is proposed and applied in a variety of different size environments. The evaluation process results show high success rates of the four approaches; however, the approach with the highest success rate is adopted. Finally, based on the results of the evaluation process, a novel algorithm is proposed to increase the efficiency of the selected approach to the optimality in the construction process of the trajectory.

## 1. Introduction

In recent years, multirotor Unmanned Aerial Vehicles (UAVs) have been getting considerable attention from many, whether researchers or ordinary people due to many features; for instance, their unique abilities to fly in various environments, whether indoor areas or outdoor densely populated civilian areas. Moreover, a unique capability of flying in any direction makes these flying vehicles suitable as a good research platform and in numerous real-world applications and missions. In addition to their low cost, high operability in various tasks with significant autonomy, and the replacement of humans in fundamental and hazardous activities, this makes UAVs preferably appropriate for unclean, tedious, or hazardous tasks compared to the conventional aircrafts that carry a human. In brief, UAVs are vehicles that can fly without a human pilot onboard [1]. There is a wide range of applications of UAVs, whether in military strategy or civilian applications, such as reconnaissance, archaeology, load transportation, surveillance, disaster relief, search and rescue, terrain mapping, environment protection, dangerous site inspection, as a 5G flying base station for providing wireless mobile connectivity, integrating renewable energies, etc. [2,3,4,5,6,7,8,9]. Since UAVs are ubiquitous in a variety of scenarios characterized by a high density of obstacles with different heights such as warehouses, houses, and cities, the most relevant challenge is how to plan a safe and flyable path for UAVs without collisions with obstacles. Consequently, the problem of planning a suitable and flyable path for a UAV in a cluttered environment is one of the most outstanding research areas. 

Path planning considers a significant process in robotic planning, especially in the operation of UAVs. It can be defined as a process of finding a low cost, feasible, optimal/near-optimal, shortest traversal path between two points (start and goal points) by meeting specific operational restrictions. Various restrictions should be taken into account when planning a path for a UAV, such as environmental uncertainties, acceleration and velocity restrictions, and inaccessible points in space. In addition to the inconvenience of lower battery duration, a myriad of the current research works consider the shortest distance of the UAV flight as the optimal aim to construct the optimal path. They merely think that the shortest the path, the least amount of energy will be consumed, forgetting the fact that the energy of the UAV can also be consumed during the change in direction (heading) in its flight. Moreover, further details can be accessed from the literature [9,10,11]. Planning a path for a multirotor UAV has numerous features compared to planning a path for a robot. The main dissimilarities for planning a path for a UAV are described as follows; first, the size of the UAV is small compared to space; it is usually represented in space as a moving point, which makes the pathfinding problem much simpler. Second, due to the UAV’s high speed, the UAV does not have the possibility to turn at an acute angle in their path. This is totally different from the movement of the robot, in which it can move slowly or even halt to rotate. Due to this matter, the path of the UAV should be smooth to avoid these turns [12,13]. Third, the UAV has the ability to alter its flight altitude (for instance, there is a UAV flying in a cluttered environment, and sometimes there is a need to change its altitude to avoid obstacles) compared to the robot that commonly moves on the earth’s surface. Therefore, the issue of planning a path for a robot is usually defined on the plane [14,15,16]. The three aforementioned features of planning a path for a UAV have been considered in this paper. 

Interestingly, the growing interest and rapid development in planning the path for UAVs, as well as data stitching, has led to the emergence of several auxiliary desktop applications, such as pixel4D and drone2map that facilitate this process in terms of processing the collected data by lightweight, UAV or aircraft. For instance, the pixel4D high-resolution capabilities allow for retrieving the elevation and even the temperature value of each pixel of taken pictures to create highly accurate maps. In addition to Drone2Map that allows the user to process imagery from commercial drones and produce 2D, 3D products such as orthoimagery and 3D meshes that easily integrate with the ArcGIS platform. It also provides workflows for inspection of critical infrastructure and monitoring of situations. These provide significant cost and time savings over conventional survey methods, producing information on the same day as imagery collection. The ArcGIS platform, in turn, allows for the sharing of the information across organizations immediately [17].

In view of the abovementioned importance of studying path planning in UAVs, it is imperative when UAVs fly in cluttered environments that they have to proceed with their task while adhering to the operational restrictions and ensuring safe navigation concerning the encompassing objects. Different approaches to UAVs path planning and for various techniques of measurement employed by the UAVs are demonstrated in [18,19,20,21,22,23,24]. In addition, several inclusive reviews for path and trajectory planning were conducted for UAVs [25], ground vehicles [26], and mobile robots [27,28,29]. While the methods may be classified in a different manner into categories, several approaches employed fall within the “grid-based” definition for flying vehicles. The path is constructed on the top of the environment’s nodes, in which this environment is discretized into a regular grid, where some of these nodes represent the free areas, which allow the transit, whereas if they restrain the transit they represent the obstacles. 

For planning a path, several grid search algorithms have been adopted to solve this problem; the most famous algorithms are Dijkstra’s algorithm [30] and its variant A* algorithm [31]. Although many years have passed since their introduction, they are still frequently in use and no research work is free of their existence, in addition to the emergence of many new algorithms that were built on their basis, especially A* algorithm. These algorithm developments include Lifelong Planning A* (LPA*) [32], Focussed D* [33], Theta* and Angle-Propagation Theta* (AP Theta*) [34,35], whereas, the alternative version of Theta* algorithm is Basic Theta* that includes Lazy Theta* [36], Phi* and Incremental Phi* [37]. 

Due to the large number of algorithms that are incorporated into solving the path planning problem, the choice was made on three of any-angle pathfinding algorithms in this paper, which are Basic Theta*, Lazy Theta*, and Phi* algorithms. The selection of these algorithms is motivated by their short runtime and paths, their capability to create superior paths that are more realistic looking and close to the actual paths, and with fewer numbers of unnecessary heading changes and waypoints. Consequently, the paths of these algorithms are more proper for smoothing and make them more congruent with the motion of the UAVs. Above all, research work is scarce for generating smooth trajectories using these algorithms because most researchers dealt with other algorithms such as A* algorithms. Therefore, these three algorithms still have room to explore by smoothing their generated paths to create collision-free trajectories. 

In general, the created grid-defined paths encompass a series of segments, which are determined by acute turns unsuitable for the aircraft flight. Due to this limitation, a model solution is presented by defining smoothing curves, which are well suited with the UAVs’ flight that allows for compelling their kinematic restrictions, where a smooth trajectory has been created by parametrizing the time of the curve [38]. The construction of the path smoothing is carried out with different mathematical curves, usually polynomial, clothoid, or spline curves [26]. The use of convex optimization and successive convex approximation (SCA) have been applied to optimize the path [39,40]. Furthermore, the curves of Bézier, which are parametric curves are repeatedly utilized in computer graphics, animation, industrial design, modeling, and many other related fields. Moreover, these curves can be applied efficiently in the robotics scope. As a matter of fact, these curves ensure considerable flexibility and low cost of the computational processes due to the fact that Bézier curves are based on polynomials shaped with the utilize of suitable control points. Therefore, Bézier curves take their role to generate a continuous smooth path; consequently, numerous applications exist of Bézier curves for flying and ground vehicles (see references [41,42,43,44]). For example, a generated path from a parallel genetic algorithm for Multi-UAV systems was smoothed by utilizing Bézier curves [45]. A dynamic collision-free trajectory was designed for UAVs by applying a six-order Bézier curve [46]. 

In this paper, a flyable and smooth path (trajectory) was constructed for a multirotor UAV. First, a feasible and suitable path is generated by using a modified any-angle path search algorithm that adopts a modified cost function. The modification includes considering the elevation of the grid map nodes that the multirotor UAV may encounter while flying from the starting location to its destination. After that, Bézier curves have been exploited for constructing a smooth curve (trajectory) that makes the UAV fly smoothly without being bounded to the acute turns of the grid-defined path. Four Bézier curves-based approaches were presented to smooth the generated paths from the pathfinding algorithms. Subsequently, an innovative evaluation process is applied to determine the most efficient approach of the four proposed approaches in a variety of different size environments. Then, the evaluation process is repeated to guarantee that the generated trajectory from the previous evaluation process is safe to be adopted. Finally, a novel algorithm is introduced to cope with the preferable approach of the proposed Bézier curves approaches directly to conduct it to the optimum state during the trajectory generating process. 

This paper is arranged as follows: Section 2 presents the strategy of path planning. Next, the trajectory design is described in Section 3. After that, the results and discussion are illustrated in Section 4. Finally, the conclusions of the research are in Section 5.

## 2. Path Planning

Path planning is a process of generating a feasible and suitable path in a cluttered environment for a UAV from a specific start point to a specific goal point. In this study, the path of the flight is created with Basic Theta*, Lazy Theta* and Phi* algorithms within a 2D grid environment. Additionally, the definition of the grid and its data structure is primarily demonstrated in this section. Subsequently, the three any-angle pathfinding algorithms and the modified cost function are described.

### 2.1. Grid Representation

**Figure 1:**
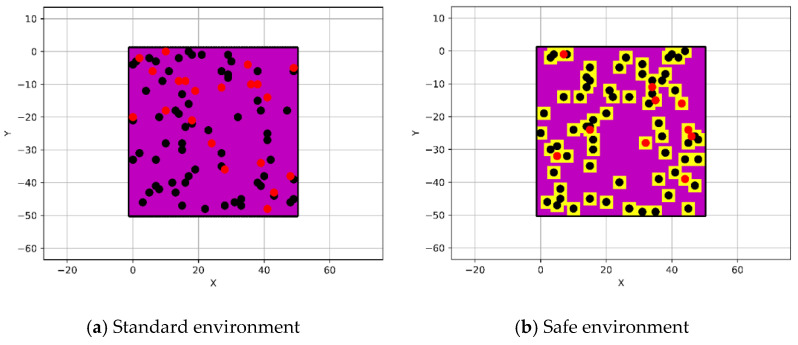
The configuration of the cluttered environment.

This section explains the properties of the environment upon which the pathfinding algorithms were implemented by creating a simple grid (binary coded) with dimensions of 50 × 50 nodes, which represents a known cluttered environment as shown in Figure 1a. The grid (a simple matrix with 50 × 50 dimensions) is used to represent the environment, where it is occupied with obstacles that are marked as 1, whereas the free spaces are marked as 0. Furthermore, the obstacles were randomly distributed over the grid with discrete values from 0 to 100, which represent their elevations, in addition to the change in their locations and elevations at each new search of the algorithms. Compared to other representation methods, the grid representation method is simple in structure and easy to implement, so it is commonly used as a way to represent the environment for path planning. To increase the integrity of the path, a safety feature (safe zone) with yellow color was augmented around the obstacles as shown in Figure 1b. The elevation of the grid map nodes, which represents E, is classified into three categories, and the severity of their danger on the UAV during the flight is relative to the designated flight altitude of the UAV, which is set to 60 during the implementation of the algorithm “drone_altitude = 60”. The elevation of the gird map nodes is set as follows:

1. E < 50. These nodes were highlighted in magenta color. These nodes represent the safest elevation for the UAV to fly above taking into consideration the UAV’s flight altitude.
2. 50 < E < 59. These nodes were highlighted in red color. They are defined as a risky elevation, which is close to the flight altitude of the UAV and it is preferred to be avoided unless there is another path to be traversed, so it will be used to fly above.
3. E > 60. These nodes were highlighted in black color. They can be avoided by the UAV while it is trying to arrive at its goal by turning around them.

### 2.2. Any-Angle Pathfinding Algorithms

**Figure 2:**
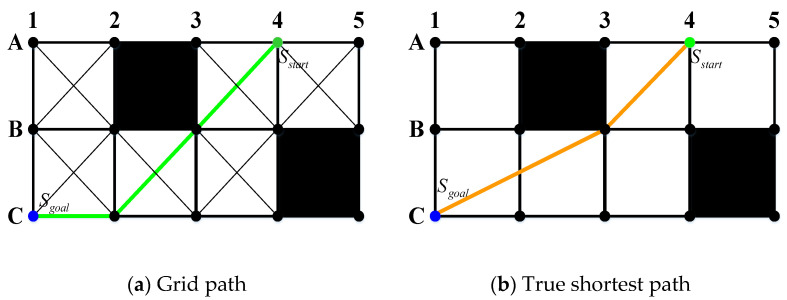
Standard find-path algorithm vs. any-angle find-path algorithm paths.

Any-angle pathfinding algorithms are developed to address some of the issues in the standard pathfinding algorithms such as A* algorithm by achieving a short unblocked path from a specific initial point to a specific target point with less computational time. Therefore, one of these issues of the standard pathfinding algorithms that have been addressed by these algorithms is the dissemination of information along graph borders and restrict paths to be constructed by these borders, as shown in Figure 2a. As a result, this restriction makes the resulting paths long and unrealistic looking compared to the actual shortest paths. For that reason, Nash and Koenig [47] developed any-angle algorithms, which disseminate information along graph borders, without restricting paths to be constructed by these borders. They can be utilized to briskly find short paths while retaining the characteristics of the standard algorithms, as shown in Figure 2b. Any-angle algorithms are based on A* algorithm which are namely Basic Theta* [34,35], Angle-Propagation Theta* (AP Theta*) [34,35], Lazy Theta* [36] and Phi* and Incremental Phi* [37] algorithms.

#### 2.2.1. Basic Theta* Algorithm

Basic Theta* algorithm is an alternative version of A* algorithm that disseminates information along graph edges without restricting the paths to be constructed by graph edges [34,35]. Additionally, it allows the direction of the path to be changed at any angle. Consequently, the motivation for utilizing the Basic Theta* algorithm is the capability of generating paths that are shorter and more realistic-looking paths than the resulting paths from the standard algorithms. Basic Theta* performs a line of sight checking, where if there is a free line of sight between two nodes, the path skips the neighbor nodes, otherwise, it traverses across the adjacent nodes as A* algorithm. Thus, the essential difference between Basic Theta* and A* algorithms is that Basic Theta* algorithm allows the parent of a node to be any node, unlike A* algorithm where the parent must be a visible neighbor. This algorithm is correct, complete, and marginally slower than A* algorithm.

#### 2.2.2. Lazy Theta* Algorithm

Lazy Theta* algorithm is the second example of any-angle pathfinding algorithms and a different version of Basic Theta* algorithm that highlights some of the deficiencies in Basic Theta* algorithm in the issue of the line of sight check when the dimension of the environment gets larger. This algorithm adopts a different idea in the matter of line-of-sight testing that differs from its predecessor; that is, it does not perform a line-of-sight test unless it is necessary. Therefore, the number of checks will be reduced, thus making it faster than the Basic Theta* algorithm. This technique is called a lazy evaluation technique, where lazy indicates the name of the algorithm itself [36]. The algorithm is correct, complete, and faster than its predecessor.

#### 2.2.3. Phi* Algorithm

Phi* algorithm is another version of Basic Theta* algorithm, which is more convenient for working in unknown environments compared to the aforementioned algorithms, especially with its incremental version. It could be incremental as a result of its concentration on some of the insufficiency in its predecessor by preserving the feature of Local Parent Path [37]. Therefore, it is described as an incremental algorithm, which means it does not need to recompute the path from scratch by each newly blocked cell, where it reuses information from preceding searches to accelerate the next one. 

The main difference between Phi* algorithm and its predecessor is that Phi* algorithm disseminates angle ranges to restrict the directions and utilizes them to locate whether two nodes have a line of sight or not. In addition, the angle range feature is employed to determine the best local parent on the path. Local parents are saved for additional utilization and are for further alteration of the path due to the fact that, on each new unexpanded visible neighbor node is carried out the line-of-sight checking. Furthermore, Phi* algorithm preserves two further values for every node, which are called lb-lower and ub-upper angle bounds, where together they form the angle range of the node [lb,ub]. Moreover, it comes up with a good tradeoff to the runtime of the search and the length of the resulting path. Also, it can find paths one order of magnitude faster than repeated Basic Theta* searches while finding paths with nearly the same lengths. The algorithm is characterized by its suitability for known and unknown environments, especially with its incremental version. It is correct, complete, and faster than the Basic Theta* algorithm. 

The common and key elements associated with the three aforementioned pathfinding algorithms when they search for a path are the cost function, open list, closed list, and the line of sight. The open list is sometimes known as the open table that stores the nodes that have not been tested yet, whereas, the closed list is sometimes known as the closed table that stores the nodes that have been tested or explored. The cost function ($f_{Any-angle})$ is the evaluation function of the shortest path from the start node, across the current node that the algorithm on, up to the goal node. The line of sight is a smart mechanism that inspects if there is a free straight line between two nodes that are not adjacent. This technique has been utilized to loosen the restriction over the resulting path to be formed by grid edges by the standard algorithms such as A* algorithm. This is because the standard algorithms only allow the motion between neighbor nodes. For a more inclusive demonstration of the algorithms, interested readers could check the following references [34,35,36,37,47].

### 2.3. Cost Function

The cost function ($f_{Any-angle})$ is the current evaluation of the shortest path from the start node, across the current node that the algorithm on, up to the goal node. Similarly to what was implemented with A* algorithm in [48], a modified cost function has been employed within the scope of this work, which targets the flying vehicles by considering the elevation of the grid map nodes. The starting point $P_{start}$ and the goal point $P_{goal}$ will be connected through defining a set of waypoints by the three pathfinding algorithms. These waypoints are selected among the available nodes of a discretized environment. Initiating from $P_{start}$, the algorithms generate a group of paths that will be expanded more and more as the algorithms continue their search for a suitable path to $P_{goal}$, until one of these paths will be adopted as the final path. The aforementioned algorithms follow two paths which are path1 and path2 or they are known as a constrained path and unconstrained path to connect two points, where if there is a free straight line between two evaluated nodes, the algorithms will connect them, otherwise, they traverse across an adjacent node similar to A* algorithm search.

The evaluation function ($f_{Any-angle})$ can calculate the valuation of all the nodes, which can search in the next step, then choose the minimum to be the next node. So, this path is the best. Consequently, it can be calculated according to Equation (1).

(1) $$f_{Any-angle}=k_{g}G+k_{h}H+k_{e}E$$

where G is the actual distance of moving from the start node to the current node on the map. Whereas, H is the valuation distance (heuristic estimated cost) from the current node to the target. The pathfinding algorithms in this paper depend on Euclidean distance for calculating H, as shown in Equation (2). Also, E represents the cost of the risk that is associated with the elevation of the node. The parameters $k_{g},\;k_{h},\;k_{e}$ represent the weights to tune the characteristics of the path and allow it to reach the goal point. The set of the parameters $k_{g}=\;$0.6, $k_{h}=$1, $k_{e}=$ 1.8 through the implementation of the algorithms is observed to guarantee a proper compromise between the numbers of the extended nodes.

(2) $$H=\sqrt{{\left(x_{j}-x_{i}\right)}^{2}+{\left(y_{j}-y_{i}\right)}^{2}}$$

where $x\left(x_{i},\;y_{i}\right)$ and $y\left(x_{j},y_{j}\right)$ represent two points in a 2D space.

## 3. Trajectory Design

Trajectory planning is the process of finding a smooth and continuous curve to move along the path. Therefore, the trajectory is a path parameterized by time. Designing a trajectory usually indicates to the matter of getting the solution from the algorithms of pathfinding and defining how to navigate along the path. First, this section explains Bézier curves and then discusses the limitation of UAVs flying in sharp turns by presenting several Bézier curves-based approaches to solve this matter.

### 3.1. Bezier Curves

**Figure 3:**
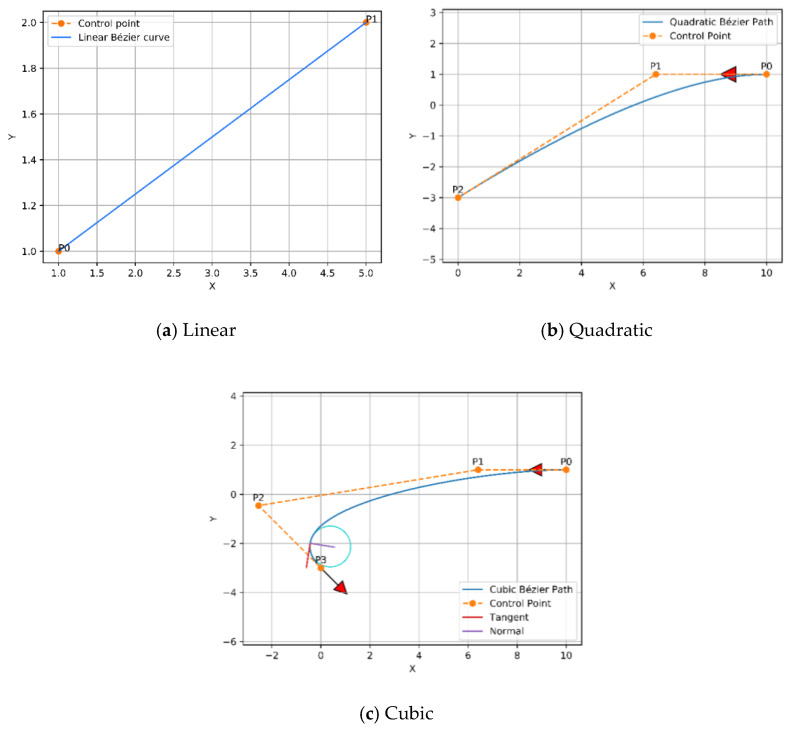
Examples of Bézier curves.

In 1962, the French engineer Pierre Bézier discovered a method of defining curves by control polygons. Based on the principle of approximation, this method constructs a set of special polynomial basis functions, which can effectively describe mathematical curves or sketches drawn by designers. Therefore, Bézier curves are parametric polynomial curves defined by the control points that are considerably employed in computer graphics to create smooth curves. The most frequent examples of Bezier curves applied are linear, quadratic, and cubic, as shown in Figure 3, in order to avoid complex computation and derivation of the high order curves. 

As previously mentioned, the definition of Bézier curve strictly depends on the number of control points of the curve. n+1 can define the curve of degree n polynomial. The parameter equation of each point on Bézier curve is as follows:

(3) $$P(t)=\sum_{i=0}^{n}P_{i}B_{i,n}(t),\;t\in [0,1]$$

where $P_{i}$ are the control points and $B_{i,n}$ is the Bernstein basis function, and its polynomial expression is as follows:

(4) $$B_{i,n}(t)=C(n,i)t^{i}{\left(1-t\right)}^{n-i},\;i=0,1,2\cdots ,n$$

where $\left(n,i\right)=\frac{n!}{i!\left(n-i\right)!}$, by substituting in Equation (3), the Bernstein basis function can be rewritten as follows:

(5) $$B_{i,n}(t)=\frac{n!}{i!(n-i)!}t^{i}{\left(1-t\right)}^{n-i}$$

The curves of Bézier always pass through the endpoints, which are the first and last control points $P\left(t=0\right)=P_{0}$, $P\left(t=n\right)=P_{n}$, whereas, the lines that connect $P_{0}$ to $P_{1}$ and $P_{n-1}$ to $P_{n}$ are tangent to these curves [44]. To design the trajectory, the waypoints of the three modified any-angles pathfinding algorithms paths are endorsed as control points of the curve. Since the environment is filled with obstacles, a safer flight of UAV is achieved by finding a midpoint between each of the two control points on each segment of the constructed path, and the waypoints with the midpoints will be connected by curves. As a result, the constructed trajectory will be constituted of several joined curves. Linear and quadratic Bézier curves were employed to design the trajectory, which are shown in Figure 3 and described in Equations (6) and (7), respectively.

(6) $$P(t)=(1-t)P_{0}+tP_{1}$$

(7) $$P(t)={\left(1-t\right)}^{2}P_{0}+2t(1-6)P_{1}+t^{2}P_{2}$$

### 3.2. Proposed Approach

As previously mentioned in Section 3.1, the most frequent examples of Bézier curves have been utilized (linear, quadratic, and cubic curves) to avoid the monotonous and exhausting computation processes. In the same section, it is also mentioned that constructing these curves strictly depends on the control points, which are the waypoints of the generated paths. It is evident that the constructed paths from the aforementioned pathfinding algorithms will contain numerous waypoints, and therefore there will be many control points that require massive mathematical operations. In fact, the curves of Bézier guarantee a significant degree of resiliency and the cost of calculation is insignificant, as they are based on polynomials that were created utilizing the appropriate control points. If the degree of the curves has risen, the previous characteristics commence disappearing when the control points number is large. A proper solution has been introduced by finding a midpoint between each two control points on each segment of the constructed path. The endpoints and control points of the curve will be connected with these midpoints by adopting quadratic Bézier curves. As a result, the final trajectory will be constituted of several joined curves. This method will guarantee an uncomplicated and efficient way to draw a curve for an innumerable number of control points. This method is achieved by using an array of points, where the points are inserted in this array and then passed to the function, thus intense and complicated mathematical operations will be avoided. 

**Figure 4:**
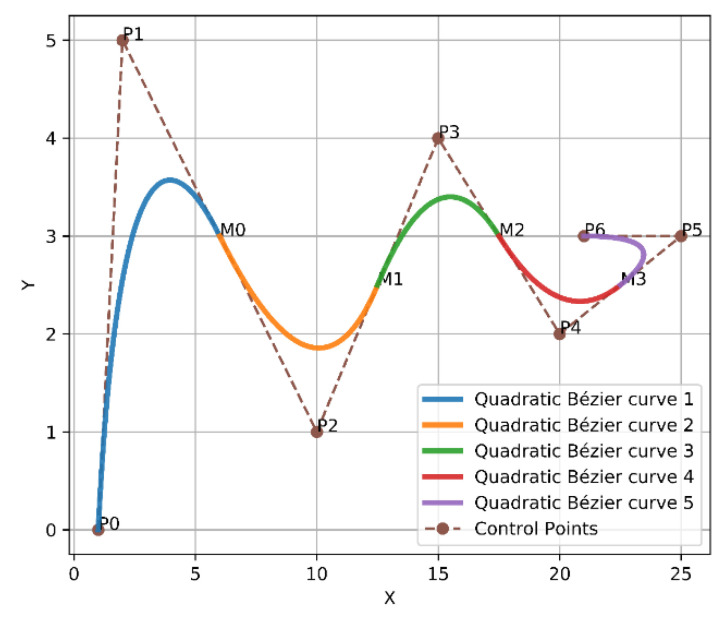
Bézier curves approach 1.

For example, Bézier curve has the following points $\left[P_{0},P_{1},P_{2},P_{3},P_{4},P_{5},P_{6}\right]$, where $P_{0}$ and $P_{6}$ represent the endpoints. Whereas, $P_{1}$ and $P_{2}$ represent the first two control points, while $P_{2}$ and $P_{3}$ represent the second two control points and so on. To apply the above solution, a first midpoint is called $M_{0}$ and is placed between the first two control points to create [$P_{1}$, $M_{0}$, $P_{2}$]. Next, a second midpoint $M_{1}$ is placed between the second two control points to form [$P_{2}$, $M_{1}$, $P_{3}$], and so on. Subsequently, from the first endpoint $P_{0}$ towards the first midpoint $M_{0}$, the first quadratic Bézier curve will be formed. After that, from $M_{0}$ to $M_{1}$, the second quadratic Bézier curve will be created as well. The two previous quadratic Bézier curves will force $C^{2}$ derivative continuity as a result of these points $\left[P_{1},M_{0},P_{2}\right]$ being in perfect alignment. The first and second derivatives of Bézier curves according to t are presented in Equations (8) and (9). Finally, this way will continuously follow the same previous approach until arriving at the final endpoint. Thus, from the first endpoint to the final endpoint, a smooth curve was constructed, as shown in Figure 4.

(8) $$\dot{P}(t)=\sum_{i=0}^{n-1}\left(P_{i+1}-P_{i})nB_{n-1,i}(t\right),$$

(9) $$\ddot{P}(t)=\sum_{i=0}^{n-2}(P_{i+2}-2P_{i+1}+P_{i})n(n-1)B_{n-2,i}(t).$$

**Figure 5:**
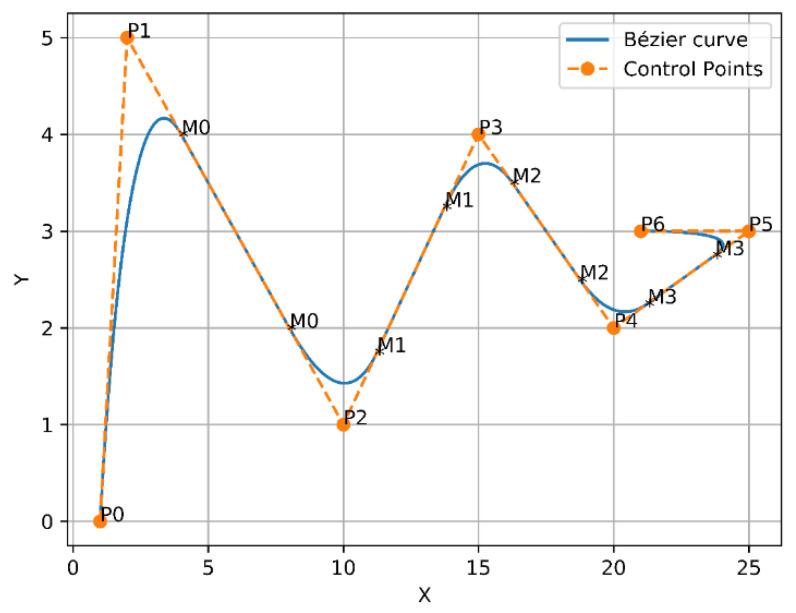
Bézier curves approach 2.

A new approach was presented by placing a couple of midpoints between each two control points on each segment of the generated path. This will add more control to the generated trajectory, as the midpoints were placed in [0.25, 0.75] positions, as shown in Figure 5.

**Figure 6:**
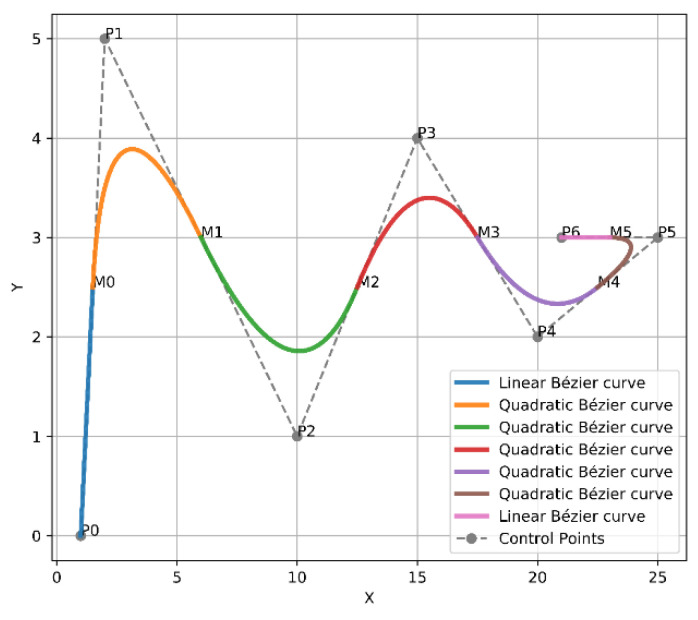
Bézier curves approach 3.

To guarantee more safety to the UAV on the end extremities of the trajectory, an additional single midpoint was placed between the endpoints and control points, as shown in Figure 6. As previously mentioned in Section 2.1, the obstacles are randomly located in the environment, that is, there is no full control on how they will behave or where they will be on the next execution to the algorithm. This may present the possibility of some obstacles close to the resulting trajectory at the final points, which could add danger to the flying vehicle. Therefore, adding a midpoint on the final extremities means adding more control to the trajectory by pushing it towards the path. In this approach, linear Bézier curves were also exploited to connect the final endpoints of the curve with the midpoints. This is because of the final extremities points of the trajectory’s just two points, which are $\left[P_{0},M_{0}\right]$ and $\left[M_{5},P_{6}\right]$, and it is inadmissible to utilize quadratic Bézier curves to represent them.

**Figure 7:**
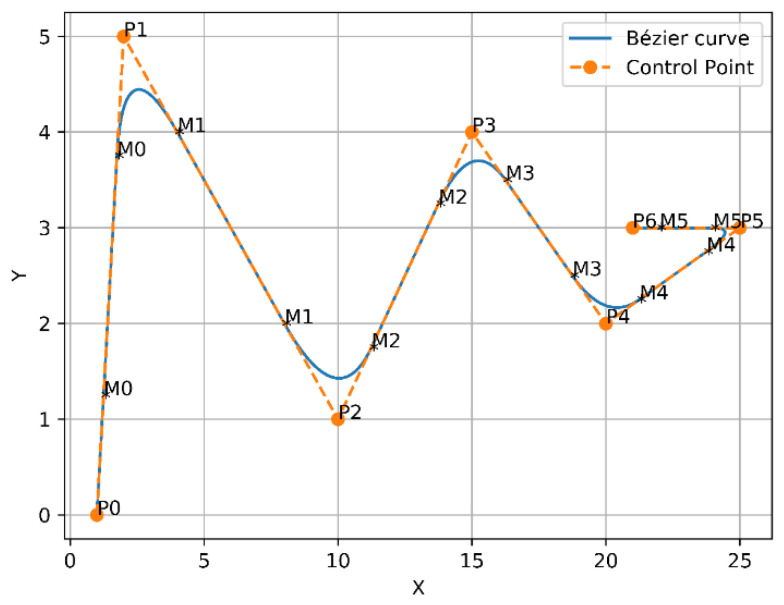
Bézier curves approach 4.

**Figure 8:**
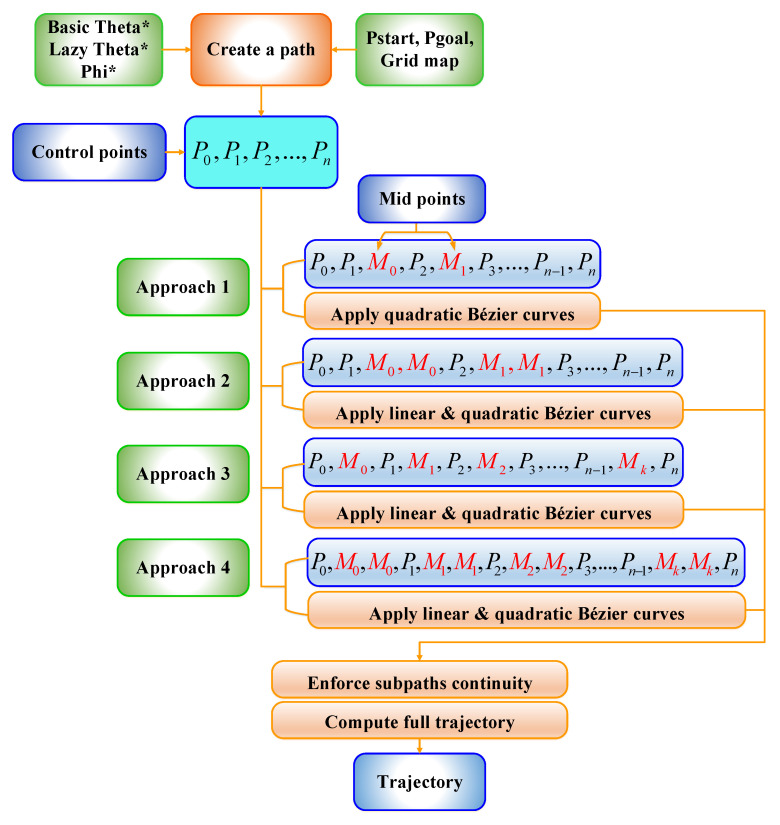
Bézier curves-based approaches.

Combining the idea of the second and third approaches, a final and new suggestion has been presented by inserting two midpoints over the whole segments of the generated path, since they were placed at the same positions of the second approach [0.25, 0.75] positions of each segment of the path, as shown in Figure 7. The addition of more points should take into account the characteristics of the generated trajectory as ensuring short, small curvature, and flyable trajectories, when it pulled out towards the path for generating more safety to the aircraft. Figure 8 clarifies the four Bézier curves-based approaches.

## 4. Results and Discussion

This section displays the results of three modified any-angle pathfinding algorithms (Basic Theta*, Lazy Theta*, and Phi*) implementation and the aforementioned four Bézier curves-based approaches over the resulting paths from the pathfinding algorithms. Additionally, it presents an innovative evaluation process of these four approaches with its results to select the near-optimum one among them in designing a flyable and collision-free trajectory with the safety feature. Simultaneously, it illustrates the re-evaluation process of the above method with its results without the safety feature. Finally, this section is concluded by presenting a new algorithm with its results that pushes the selected approach to the optimality in constructing the trajectory. 

### 4.1. Implementation of the Modified Any-Angle Pathfinding Algorithms

**Figure 9:**
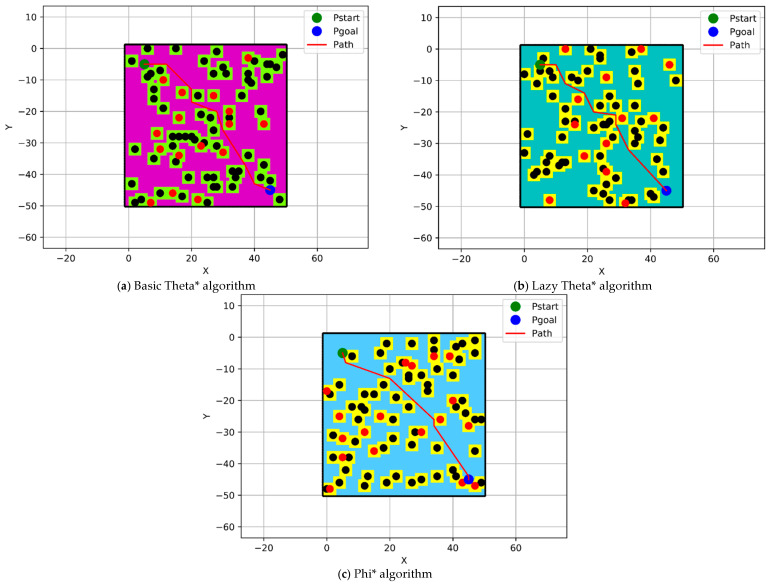
Modified any-angle pathfinding algorithms paths.

The unblocked paths of the modified any-angle pathfinding algorithms, which are Basic Theta*, Lazy Theta*, and Phi*, have been displayed with red color in this section, as shown in Figure 9. The positions of the start and goal points have been located at (5,5) coordinates with a green color and (45,45) coordinates with a blue color within a grid size of 50 × 50 nodes. For more clarification, the color of the nodes indicates that their elevation less than 50 is changed from one algorithm to the other.

### 4.2. Performance Comparison of the Three Any-Angle Pathfinding Algorithms

A comparison has been made among the aforementioned any-angle pathfinding algorithms in terms of runtime, where it is intended to know how long each algorithm takes while searching for an unblocked path between the starting and goal points. Time plays an important role in pathfinding algorithms’ work and the faster the algorithm is, the more appropriate it is to be adopted, especially in larger environments and particularly in some current applications that depend considerably on pathfinding algorithms. The runtime is calculated by using Python programming code for each algorithm where the code was executed 100 times and the average value has been taken. All the algorithms were applied to a 2D environment with two different grid sizes (size 1 = 50 × 50 and size 2 = 100 × 100). Furthermore, the start and goal points have been set to ($x_{start}=5$,$\;y_{start}=5$), ($x_{goal}=45$,$y_{goal}=45$) for a grid size 50 × 50 and ($x_{start}=5$, $y_{start}=5$), ($x_{goal}=95$,$\;y_{goal}=95$) for a grid size 100 × 100. Table 1 displays the elapsed time that has been taken by each algorithm to find the path on the environment with two different grid sizes.

It is obvious by observing the runtime of each algorithm to find a path from the initial node to the goal node based on varying the environment size; it can be observed that Basic Theta* algorithm is the slowest algorithm between the other two implemented algorithms, whereas the Lazy Theta* and Phi* algorithms have shown convergence in the runtime for finding the path, and they both are faster than the Basic Theta* algorithm.

### 4.3. Implementation of Bézier Curves-Based Approaches on the Pathfinding Algorithms

In this section, a continuous flyable trajectory for a multirotor UAV has been constructed using the four aforementioned Bézier curves-based approaches with the three modified any-angle pathfinding algorithms. The characteristics of the environment are well explained in Section 2.1. The positions of the start and goal points will be changed for each algorithm and the design of the trajectory will be located within a grid size of 50 × 50 nodes. The four proposed Bézier curves-based approaches are demonstrated as follow:

1. A single midpoint has been located at the center of each segment of the generated path excluding the first and the final segments. This approach was named the Bézier1 curve approach.
2. Two midpoints have been located at [0.25, 0.75] positions of each segment of the generated path excluding the first and the final segments. This approach was named the Bézier2 curve approach.
3. A single midpoint has been located at the center of each segment of the generated path including the first and the final segments. This approach was named the Bézier3 curve approach.
4. Two midpoints have been located at [0.25, 0.75] positions of each segment of the generated path including the first and the final segments. This approach was named the Bézier4 curve approach.

#### 4.3.1. Basic Theta* Algorithm

**Figure 10:**
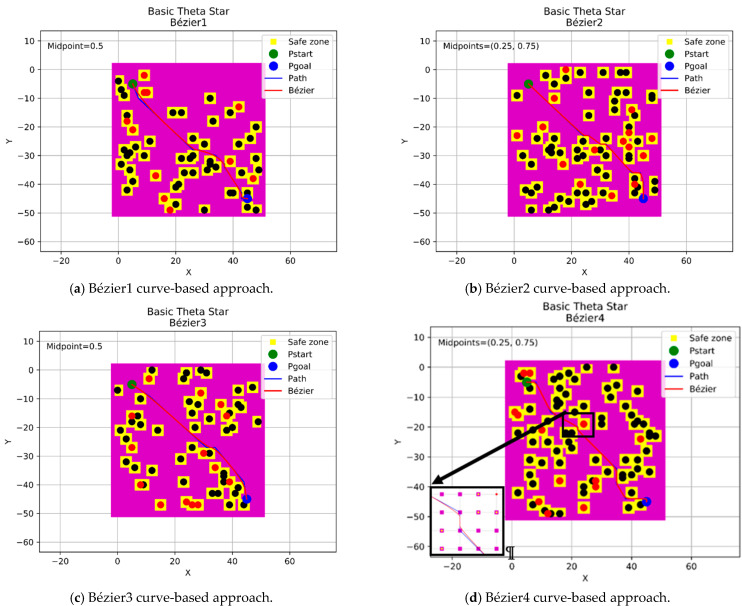
The trajectories of the four Bézier curves-based approaches with Basic Theta*.

This section displays the design of the flyable trajectories with Basic Theta* algorithm using the aforementioned four approaches, as shown in Figure 10. The positions of the start and goal points have been located at (5,5) coordinates with a green color and (45,45) coordinates with a blue color within a grid size of 50 × 50 nodes. 

#### 4.3.2. Lazy Theta* Algorithm

**Figure 11:**
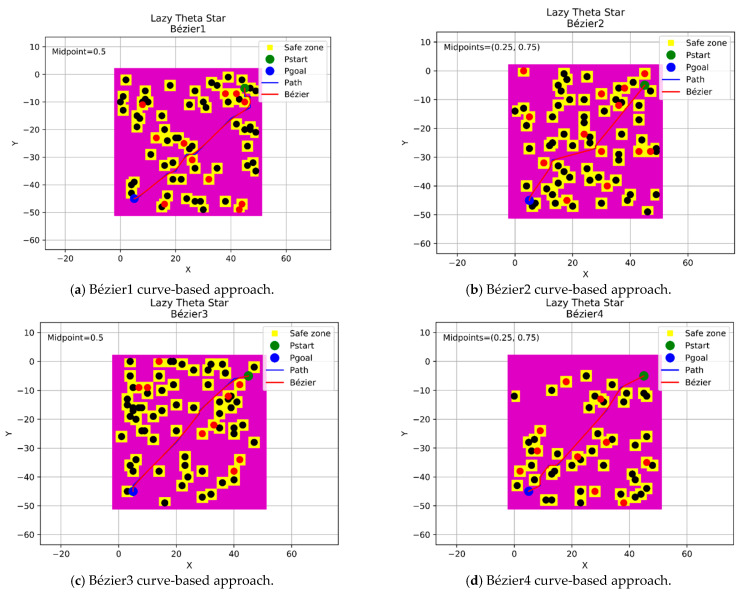
The trajectories of the four Bézier curves-based approaches with Lazy Theta*.

This section displays the design of the flyable trajectories with the Lazy Theta* algorithm using the four Bézier curves-based approaches, as shown in Figure 11. The positions of the start and goal points have been located at (5,45) coordinates with a green color and (45,5) coordinates with a blue color within a grid size of 50 × 50 nodes. 

#### 4.3.3. Phi* Algorithm

**Figure 12:**
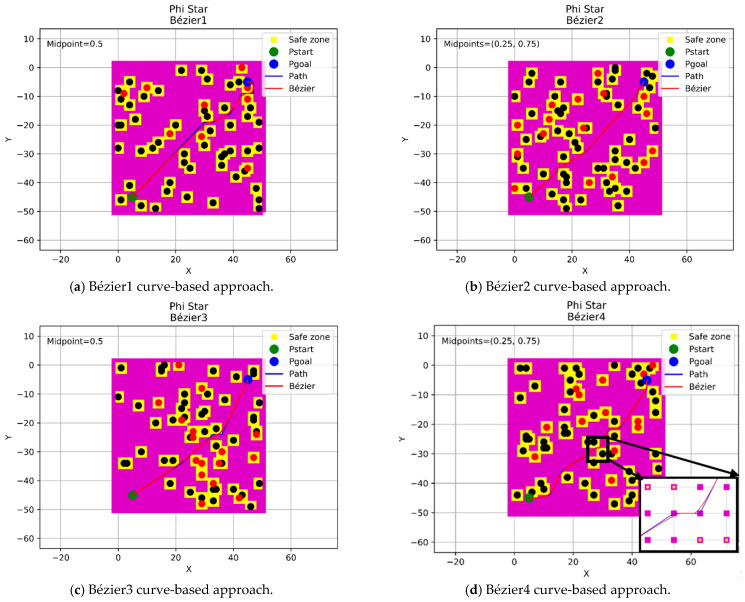
The trajectories of the four Bézier curves-based approaches with Phi*.

This section displays the design of the flyable trajectories with the Phi* algorithm using the four Bézier curves-based approaches, as shown in Figure 12. The positions of the start and goal points have been located at (45,5) coordinates with a green color and (5,45) coordinates with a blue color within a grid size of 50 × 50 nodes. 

It is obvious from the above Figure 10, Figure 11 and Figure 12 that the four approaches of Bézier curves have conducted their job in creating a flyable and smooth trajectory for the aircraft when they were applied to the generated paths of the three modified any-angle pathfinding algorithms. Simultaneously, some questions may arise. How safe and successful are these trajectories? Are they safe enough to be adopted? Are all these approaches applicable? These questions will be answered in the next sections by presenting a novel and innovative evaluation processes to validate the integrity and applicability of those approaches, additionally, specifying the near-optimal and the most suitable approach to be adopted in designing the trajectory.

### 4.4. Evaluation Process of the Proposed Bézier Curves-Based Approaches with the Safety Feature

This section answers the questions that have been asked at the end of the previous section in detail by introducing a new and innovative mechanism to evaluate the four approaches, which were studied in Section 3.2. This process aims to find a proper approach that guarantees the construction of a flyable and smooth trajectory from among the four Bézier curves-based approaches that have been proposed. The four approaches were evaluated by applying them to a variety of different size grid maps; the more environments there are, the greater the confidence in knowing whether one of these approaches is effective to be adopted. Consequently, 1000 randomly generated environments were employed to evaluate the four approaches, which are Bézier1, Bézier2, Bézier3, and Bézier4. Additionally, one of the three any-angle pathfinding algorithms in this paper was utilized in each environment beside the four Bézier curves-based approaches with randomly generated obstacles and start and goal points. Due to the research work being scarce on the Phi* algorithm and the fact that it has a lot of room to explore, it will be directly adopted in the evaluation process. After that, a validation mechanism was constructed for each operation that takes place in each environment, which includes (path and trajectory design) examining their rate of success. After generating the path and the four trajectories on each environment, this mechanism checks each point along each constructed trajectory with a rounding process.

**Figure 13:**
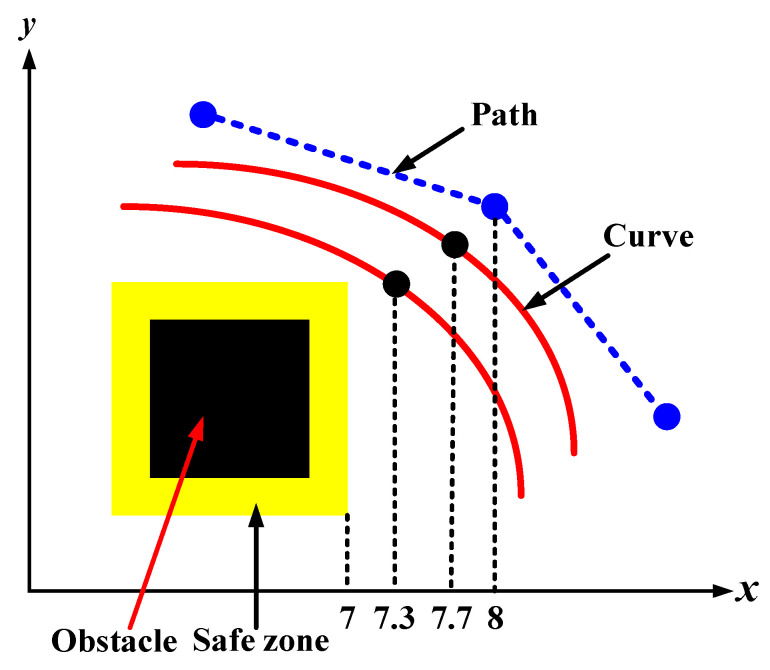
The rounding process of Bézier curve points.

For example, for a trajectory that crosses a specific point that existed in the free space between the generated path from the modified Phi* algorithm and the safety feature, which is the yellow buffer area that surrounds the obstacles, if this point exists on a 7.3 position, it is rounded to be 7, this will be considered as an inappropriate point. Whereas, if the location of this point is located on a 7.7 position, it is rounded to be 8; this will be considered a suitable point and so on. The distance between the safety feature and the path is one of the grid environment coordinates, as shown in Figure 13. The points of Bézier curves lie within the grid map, so the validator examines their positions within the grid and rounds their coordinates to check if they are close to the buffer area or the path. It may happen that a point on the trajectory does not cross the buffer area and at the same time is considered inadmissible due to the rounding process, while in reality, it could be a good point. The above approach is considered conservative. For this reason, the success rate was not found to be 100%, but it does not mean that the results are unsatisfactory because it still shows very high results of success rates of up to 89.9%. This evaluation process is only to confirm that these approaches are generating safe and smooth trajectories and even far from the safety feature. It is not about proving that Bézier curves are proper to be adopted or not for trajectory design, since all the aforementioned approaches had the ability to create flyable trajectories.

The evaluation process is performed by creating a 4 × 1000 matrix. Each row of this matrix represents one of the Bézier curves-based approaches, while each column will include the evaluation result (success rate) for each whole operation on each environment. As a result, if the rounding process has proven that the whole constructed trajectory from the starting point to the goal point is far from the safety feature, set 1 in the corresponding matrix position, otherwise 0. 

**Figure 14:**
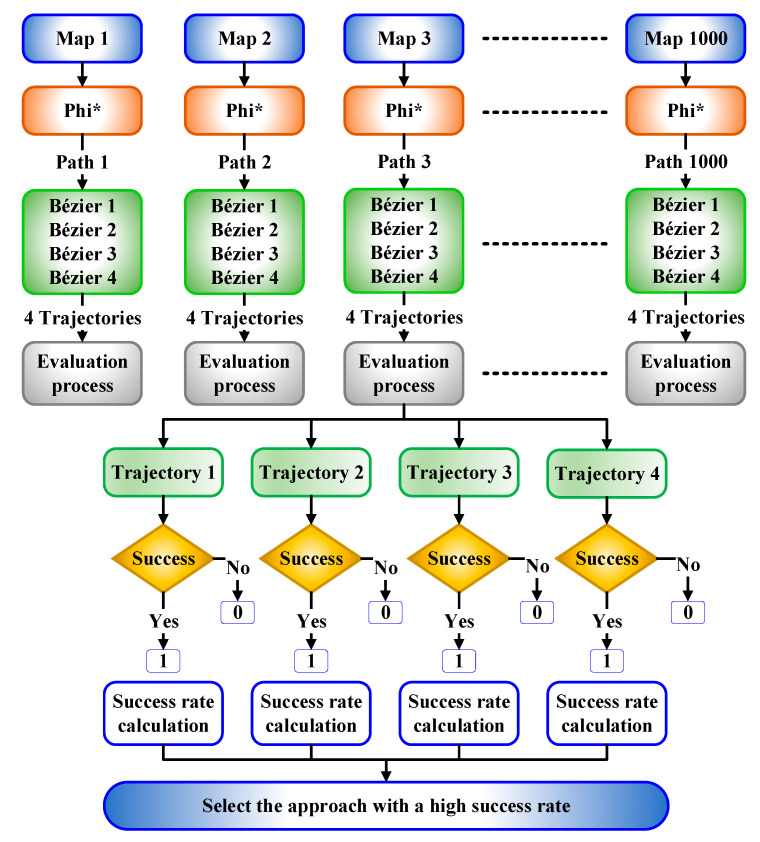
The evaluation process of Bézier curves-based approaches.

Finally, the success rate of each approach is calculated based on the number of ones in each row. The higher the number of ones within the row, the more appropriate this approach is to be adopted in creating the trajectory. The evaluation process has been implemented on a machine, which has the configurations as in Table 2. The evaluation process of the four Bézier curves-based approaches is illustrated in Figure 14.

**Figure 15:**
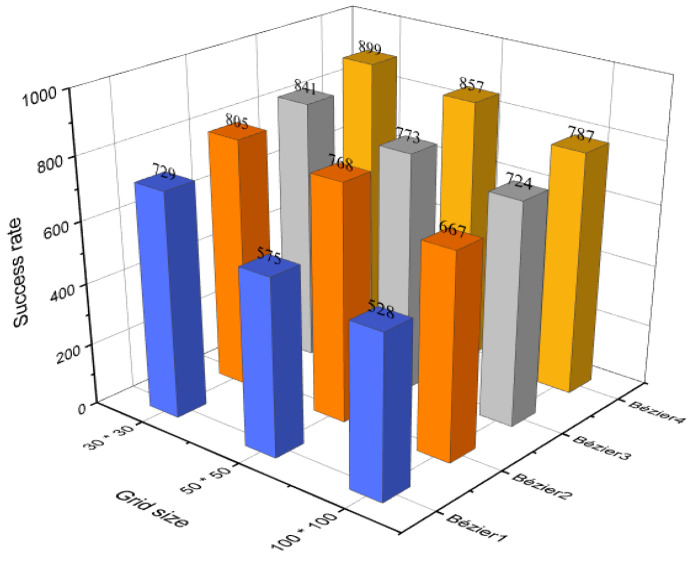
The success rate of Bézier curves-based approaches with the safety feature.

Looking at Figure 15 that illustrates the results of the evaluation process, which were applied to three different sizes of environments (30 × 30, 50 × 50, 100 × 100), it is obvious that the first approach takes the least amount of success rates, whereas, the fourth approach imposed its dominance in all cases among the others. This explains that the Bézier4 curve-based approach is a suitable approach to be adopted. It was also seen, with the increase in the size of the environments, that the success rates are decreasing, due to the fact that the generated trajectory will be long, which explains that it will encounter more obstacles, and therefore there is a higher probability to pass close to or inside the safety feature area. As a result, this evaluation process will be repeated in the next section without considering the safety feature just to guarantee the extent of the integrity of the generated trajectory.

### 4.5. Re-Evaluation Process of the Proposed Bézier Curves-Based Approaches without the Safety Feature

**Figure 16:**
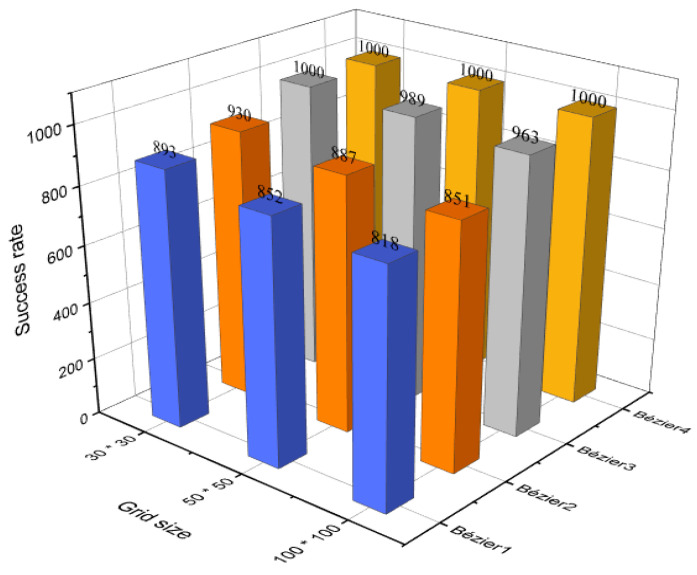
The success rate of Bézier curves-based approaches without the buffer area.

To investigate the possibility of generating collision-free trajectories, the aforementioned evaluation process of the four Bézier curves-based approaches in Section 4.4 was repeated with the same previous conditions by examining the points on Bézier curves without considering the surrounded buffer area. From Figure 16, it is clear that the Bézier4 curve approach demonstrated again its dominance with a success rate of 100% in the three different sizes of the environment. Whereas, Bézier1, Bézier2, and Bézier3 curves approaches also accentuated high success rates and they decreased gradually with the increase of the environment size due to the length of the generated trajectories and the large number of obstacles, which may cause an increase in the probability of trajectory colliding with obstacles due to the rounding process.

### 4.6. A New Approach to Increasing the Efficiency of Bézier4 Curve-Based Approach to the Optimality

**Figure 17:**
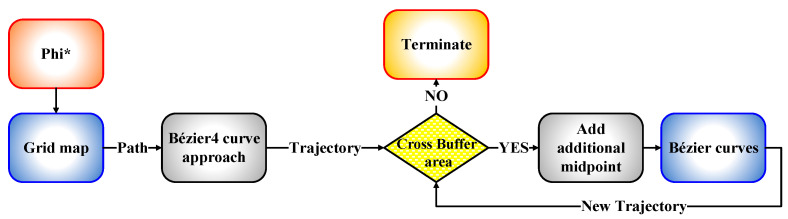
The schematic diagram of the new proposed algorithm.

After the evaluation process was conducted on the four Bézier curves-based approaches, it was found that the fourth approach is the most suitable to be adopted in designing the trajectory compared to the three other approaches, since it showed high success rates, as shown in Figure 15 and Figure 16. To reach the optimality in generating a safe and smooth trajectory, a new algorithm has been proposed to work with the Bézier4 curve approach. This algorithm suggests adding further midpoints, which guarantees to increase the safety of the generated trajectory with a very high success rate and generate an optimal trajectory far from the yellow buffer area. To illustrate the work of the proposed algorithm, a schematic diagram of the algorithm is presented in Figure 17.

**Figure 18:**
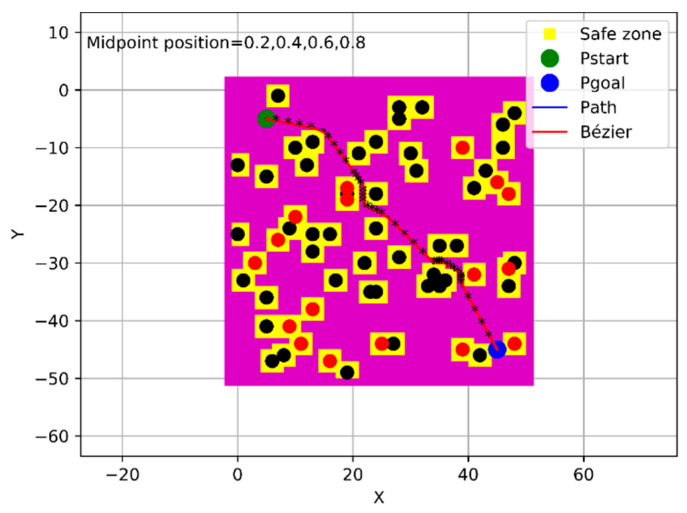
Phi* path with the Bézier4 curve approach within the new algorithm.

**Figure 19:**
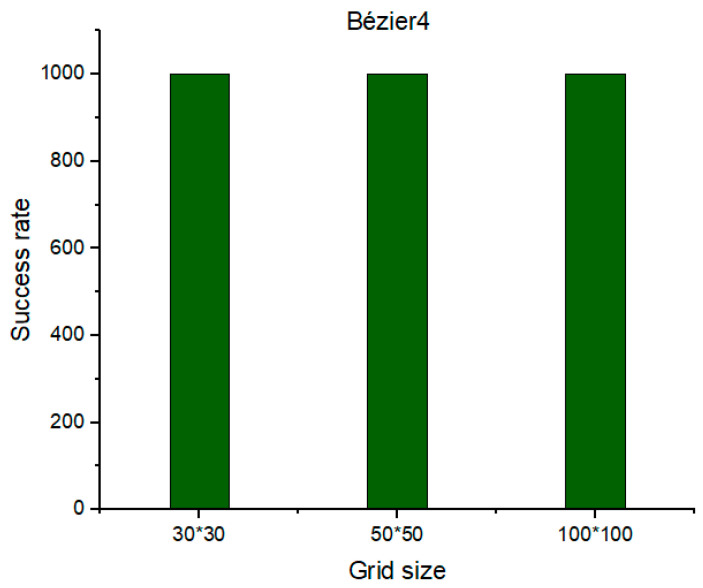
The success rate of the Bézier4 curve approach over Phi* algorithm.

From the process described above, the proposed algorithm showed its success in generating an optimal trajectory from a specific starting point to a specific goal point, as shown in Figure 18. The intended near-optimal and/or optimal criterion means the guarantee of generating a flyable path from the specific starting point to the specific goal point without colliding with the obstacles that have random and uncontrolled behavior, in terms of generation, in addition to ensuring that the safety feature that was constructed around the obstacles is not bypassed by the UAV. Therefore, the amount of this optimality was determined based on the success rates that were shown in Section 4.4, Section 4.5 and Section 4.6 which are up to 100%. The added midpoints to the generated path assured to drag the generated trajectory towards the path and keep it far from the buffer area. In addition to the previous two midpoints that are already within the fourth approach of the Bézier curve, another two midpoints have been added according to the new algorithm. Therefore, the algorithm has been iterated twice by adding a midpoint at each iteration, so that the sum of the added midpoints is four, which are distributed equally in each segment of the path in order [0.20, 0.40, 0.60, 0.80] and are illustrated with black asterisks in Figure 18. Subsequently, the evaluation process has been applied for the generated trajectory with the Bézier4 curve approach; as a result, the success rate has reached up to 100% under the three different sizes of the environment, as shown in Figure 19.

## 5. Conclusions

This paper debates how to construct an optimal and flyable trajectory for a multirotor UAV in a cluttered environment. Mainly, three algorithms of any-angle pathfinding algorithms (Basic Theta*, Lazy Theta*, and Phi*) were employed for solving the problem of path planning. The conclusion is drawn as follow:

1. Feasible paths were calculated by employing three modified algorithms of any-angle pathfinding algorithms over a conventional grid.
2. The resulting paths were characterized by angular or sharp turns that are inappropriate for the flying vehicles; therefore, the properties of Bézier curves were exploited for smoothing the path by presenting four Bézier curves-based approaches.
3. An innovative evaluation process was proposed to determine the most proper approach of the four Bézier curves-based approaches in constructing a flyable and collision-free trajectory.
4. Based on the evaluation process results, a novel algorithm was proposed to push the chosen Bézier curve-based approach to be optimal by adding further midpoints. As a result, it has proven its effectiveness by generating an optimal trajectory with a success rate of up to 100%.

In future work, the characteristics of the 3D environment are planned to be considered for application with multirotor UAVs.

## Figures and Tables

**Table 1:** Performance comparison of any-angle pathfinding algorithms.

	Grid Size	Basic Theta*	Lazy Theta*	Phi*
Time [Sec]	50 × 50	0.08818	0.03178	0.03403
Time [Sec]	100 × 100	0.1939	0.0603	0.06112

**Table 2:** The properties of the test platform.

Properties	Values
Computer	LEGION—Lenovo
CPU	Intel(R) Core(TM) i7-8750h 2.20 GHz
RAM	8.00 GB
Operating System	64 bit Windows 10 Pro edition
Programming Language	Python
IDE	JetBrains PyCharm Community Edition 2019.1.1 x64

## Data Availability

Not applicable.
